# Aqua­[6-carboxyl­ato-*N*′-(pyridin-2-yl­methyl­idene)pyridine-2-carbohydrazidato]copper(II) trihydrate

**DOI:** 10.1107/S1600536812021447

**Published:** 2012-05-19

**Authors:** Yu-Min Huang, Wen-Shi Wu, Xin-Yu Wang

**Affiliations:** a College of Materials Science and Engineering, Huaqiao University, Xiamen, Fujian 361021, People’s Republic of China

## Abstract

In the title compound, [Cu(C_13_H_8_N_4_O_3_)(H_2_O)]·3H_2_O, the complex molecule, except for the aqua ligand, is essentially planar [r.m.s. deviation = 0.034 (2) Å]. The coordination polyhedron of the Cu^2+^ cation is a square-pyramid, with the aqua ligand at the apex. The compound exhibits a three-dimensional structure, which is is stabilized by O—H⋯O and O—-H⋯N hydrogen bonds and π–π inter­actions [centroid–centroid distance = 2.987 (3) Å].

## Related literature

For the synthesis, see: Wu *et al.* (2007 ▶). For a related structure, see: Cheng *et al.* (2007 ▶).
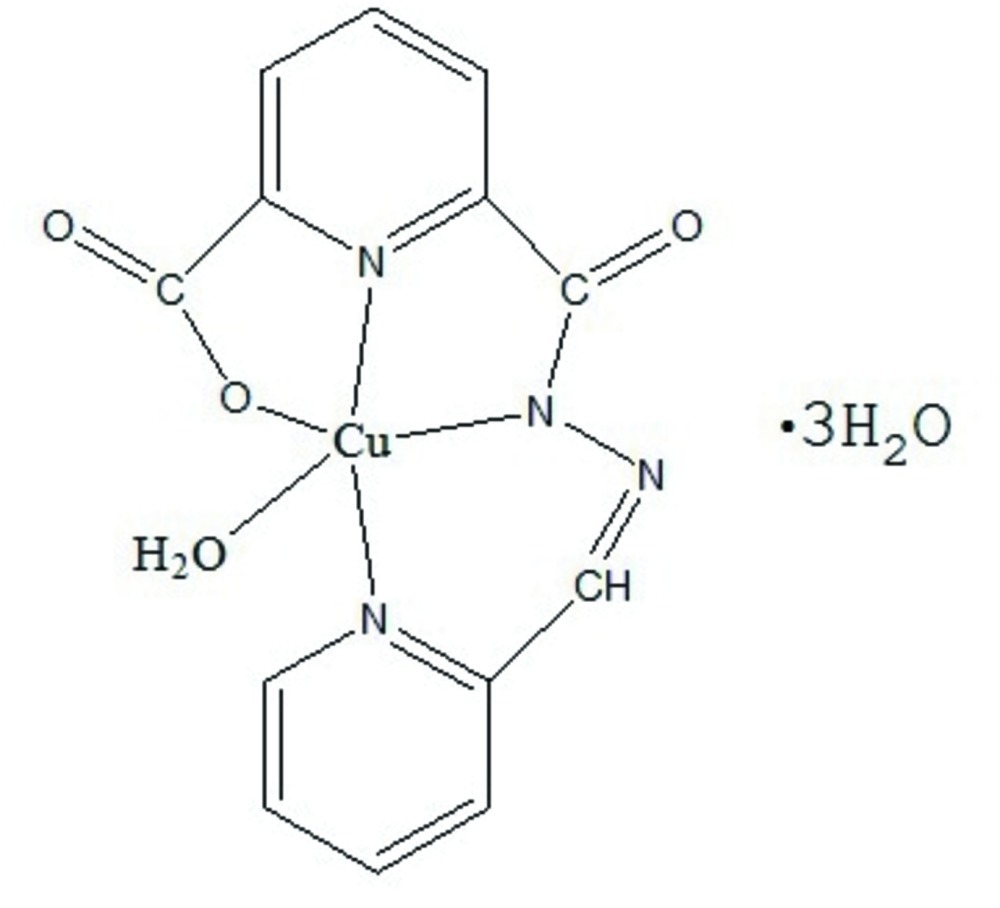


## Experimental

### 

#### Crystal data

[Cu(C_13_H_8_N_4_O_3_)(H_2_O)]·3H_2_O
*M* *_r_* = 403.85
Triclinic, 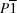

*a* = 7.1646 (16) Å
*b* = 9.369 (2) Å
*c* = 12.647 (3) Å
α = 75.313 (4)°
β = 78.864 (4)°
γ = 74.155 (4)°
*V* = 783.0 (3) Å^3^
*Z* = 2
Mo *K*α radiation
μ = 1.44 mm^−1^
*T* = 173 K
0.46 × 0.25 × 0.20 mm

#### Data collection

Bruker SMART CCD diffractometer
Absorption correction: multi-scan (*SADABS*; Sheldrick, 1996 ▶) *T*_min_ = 0.655, *T*_max_ = 0.749
4689 measured reflections
3903 independent reflections
3270 reflections with *I* > 2σ(*I*)
*R*_int_ = 0.017

#### Refinement

*R*[*F* ^2^ > 2σ(*F* ^2^)] = 0.032
*wR*(*F* ^2^) = 0.097
*S* = 1.09
3903 reflections
259 parameters
H atoms treated by a mixture of independent and constrained refinement
Δρ_max_ = 0.87 e Å^−3^
Δρ_min_ = −0.34 e Å^−3^

### 

Data collection: *SMART* (Bruker, 1999 ▶); cell refinement: *SAINT* (Bruker, 1999 ▶); data reduction: *SAINT*; program(s) used to solve structure: *SHELXS97* (Sheldrick, 2008 ▶); program(s) used to refine structure: *SHELXL97* (Sheldrick, 2008 ▶); molecular graphics: *SHELXTL* (Sheldrick, 2008 ▶); software used to prepare material for publication: *SHELXTL*.

## Supplementary Material

Crystal structure: contains datablock(s) global, I. DOI: 10.1107/S1600536812021447/hg5211sup1.cif

Structure factors: contains datablock(s) I. DOI: 10.1107/S1600536812021447/hg5211Isup2.hkl

Additional supplementary materials:  crystallographic information; 3D view; checkCIF report

## Figures and Tables

**Table 1:** Hydrogen-bond geometry (Å, °)

*D*—H⋯*A*	*D*—H	H⋯*A*	*D*⋯*A*	*D*—H⋯*A*
O4—H4*B*⋯O1^i^	0.70 (3)	2.04 (3)	2.718 (2)	165 (3)
O7—H7*B*⋯O6^ii^	0.72 (3)	2.09 (3)	2.796 (3)	167 (3)
O6—H6*A*⋯O3^iii^	0.74 (3)	1.94 (3)	2.675 (3)	176 (3)
O5—H5*B*⋯O4	0.72 (3)	2.07 (3)	2.788 (3)	173 (3)
O4—H4*A*⋯N3^iv^	0.70 (4)	2.20 (4)	2.878 (2)	163 (3)
O4—H4*A*⋯O1^iv^	0.70 (4)	2.56 (3)	3.053 (2)	129 (3)
O5—H5*A*⋯O7	0.65 (3)	2.10 (4)	2.742 (3)	168 (4)
O7—H7*A*⋯O6^v^	0.86 (4)	1.95 (4)	2.803 (3)	175 (3)
O6—H6*B*⋯O5	0.78 (4)	1.94 (4)	2.718 (3)	178 (3)

Symmetry codes: (i) 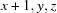; (ii) 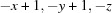; (iii) 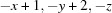; (iv) 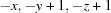; (v) 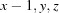.

**Fig. 1.**
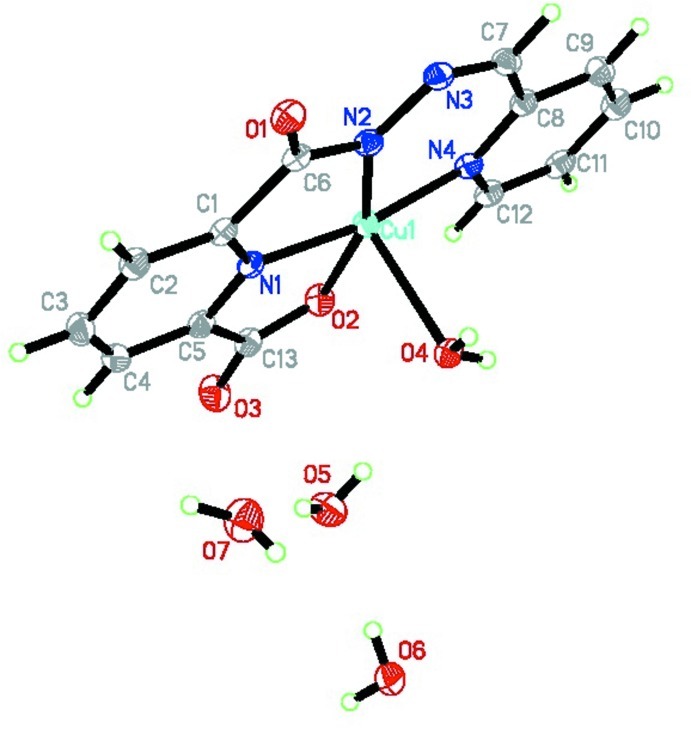
The molecular structure (at 30% probability) of the title compound.

**Fig. 2.**
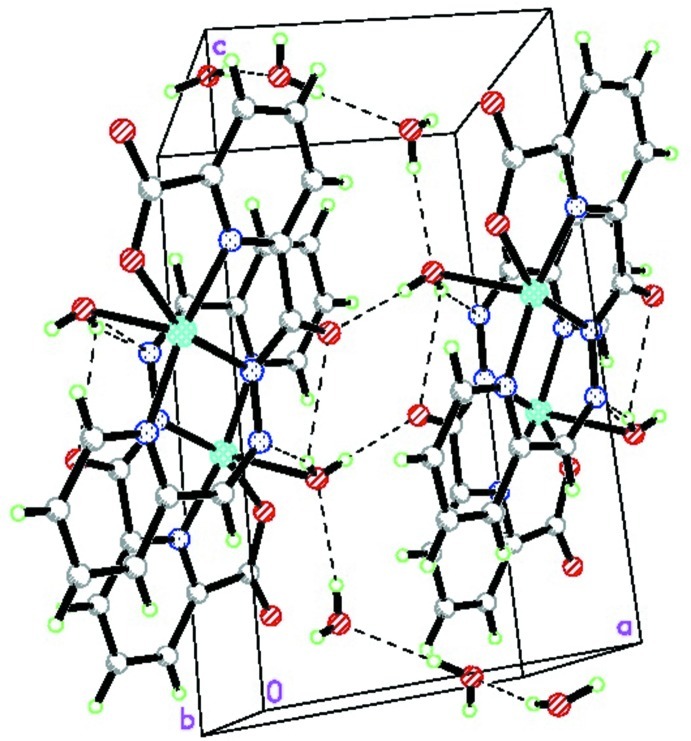
Packing diagram of the title complex, showing hydrogen bonds as dashed lines.

## supplementary crystallographic information

### Comment

In the title compound, [(C_13_H_8_N_4_O_3_)(H_2_O)Cu].3H_2_O (I), the Cu(II) ion is 5-coordinated by two nitrogen from two pyridine rings of the same molecule, one nitrogen from the hydrazine, one carboxyl oxygen, and an oxygen atom from H_2_O. They form a rectangular pyramid. N1, N2, N4, O2 from the bottom side (Rms=0.0039 (7) Å), The distances of Cu and O4 to the plane are 0.1446 (8)Å and 2.477 (2)Å. The Cu—O bond lengths Cu—O2 and Cu—O4 are 2.008 (2) Å and 2.338 (2) Å , the bond lengths of two pyridine ring nitrogens with Cu are 1.940 (2) Å and 1.932 (2) Å, which are a little shorter then the normal value(1.99 Å). The distance of Cu—N2 is 1.942 (2) Å. The structure of the title compound shown in Fig 1. Except for the H_2_O molecules and the Cu atom , the complex molecule is essentially planar, the r.m.s. deviation from planarity being 0.034 (2) Å. It exhibits a three-dimensional structure which is stabilized by hydrogen bonds, van der Waals forces and π–π interactions [the distance between the layers is 0.987 (3) Å]. The O—H···N, O—H···O hydrogen bonds are detailed in Fig 2 and Table 1.

### Experimental

Concentrated H_2_SO_4_ (2 mL)was added slowly with stirring to a solution of pyridine-2,6-dicarboxylic acid in ethanol. The solution was left to reflux for 24 h, yielding a white precipitate of ethylpyridine-2,6-dicarboxylate. This was dissolved in ethanol, then the hydrazine hydrate was slowly added with continuous stirring and the mixture was refluxed over a period of 6 h, yielding awhite crystalline solid of pyridine-2-carbohydrazide-6-carboxyl acid.

The synthesis of N^2^-(pyridin-2-ylmethylidene)-pyridine-2-carbohydrazide methylformamide -6-carboxylic acid was carried out in accord with the method of Cheng *et al.* , (2007). To a suspension of pyridine-2-carbohydrazide-6-carboxyl acid (5.43 g, 30 mmol) in absolute ethanol(50 ml), a solution of pyridine-2-aldehyde (6.43 g, 60 mmol) in the same solvent(20 ml) was added at 353 K. The mixture was left to react at refluxing for 8 h. The yellowish product was filtered, washed with hot ethanol(20 ml) three times and dried in vacuo.

The title compound (I) was synthesized according to the method of Wu *et al.*, (2004). The N^2^-(pyridin-2-ylmethylidene)-pyridine-2-carbohydrazide methylformamide -6-carboxylic acid (0.03 g,0.1 mmol) dissolved in DMF(10 ml), then CuBr_2_(0.02 g, 0.1 mmol) in DMF(10 ml) was added slowly. Black crystals of the title complex precipitated after a few weeks of slow evaporation of the DMF solution at room temperature. Elemental analysis: caculated for C_13_H_10_CuN_4_O_4_.3H_2_O:C 38.61%, H 3.96%, N 13.86% ; found: C 38.70%, H 3.83%, N 13.95%. Mp: 645 K.

### Refinement

The position of the water H atoms were located in a difference Fourier map and were refined freely. *U*_iso_ of H4A, H4B, H6A atom = 0.03*U*_eq_(C), *U*_iso_ of H5A, H5B, H7B atom = 0.03*U*_eq_(C), and *U*_iso_ of H6B, H7A atom = 0.06*U*_eq_(C). All the C-bound H atoms were included in the riding model approximation with C—H = 0.93 Å. The *U*_iso_ of each H atom = 1.2*U*_eq_(C).

### Crystal data

**Table:**

[Cu(C_13_H_8_N_4_O_3_)(H_2_O)]·3H_2_O	*Z* = 2
*M**_r_* = 403.85	*F*(000) = 414.0
Triclinic, *P*1	*D*_x_ = 1.713 Mg m^−^^3^
Hall symbol: -P 1	Mo *K*α radiation, λ = 0.71073 Å
*a* = 7.1646 (16) Å	Cell parameters from 4689 reflections
*b* = 9.369 (2) Å	θ = 2.3–28.3°
*c* = 12.647 (3) Å	µ = 1.44 mm^−^^1^
α = 75.313 (4)°	*T* = 173 K
β = 78.864 (4)°	Prism, black
γ = 74.155 (4)°	0.46 × 0.25 × 0.20 mm
*V* = 783.0 (3) Å^3^	

### Data collection

**Table:**

Bruker SMART CCD diffractometer	3903 independent reflections
Radiation source: fine-focus sealed tube	3270 reflections with *I* > 2σ(*I*)
Graphite monochromator	*R*_int_ = 0.017
ω scans	θ_max_ = 28.3°, θ_min_ = 2.3°
Absorption correction: multi-scan (*SADABS*; Sheldrick, 1996)	*h* = −9→5
*T*_min_ = 0.655, *T*_max_ = 0.749	*k* = −12→11
4689 measured reflections	*l* = −16→15

### Refinement

**Table:**

Refinement on *F*^2^	Primary atom site location: structure-invariant direct methods
Least-squares matrix: full	Secondary atom site location: difference Fourier map
*R*[*F*^2^ > 2σ(*F*^2^)] = 0.032	Hydrogen site location: inferred from neighbouring sites
*wR*(*F*^2^) = 0.097	H atoms treated by a mixture of independent and constrained refinement
*S* = 1.09	*w* = 1/[σ^2^(*F*_o_^2^) + (0.060*P*)^2^ + 0.2857*P*] where *P* = (*F*_o_^2^ + 2*F*_c_^2^)/3
3903 reflections	(Δ/σ)_max_ = 0.001
259 parameters	Δρ_max_ = 0.87 e Å^−^^3^
0 restraints	Δρ_min_ = −0.34 e Å^−^^3^

### Special details

**Table:**

Geometry. All esds (except the esd in the dihedral angle between two l.s. planes) are estimated using the full covariance matrix. The cell esds are taken into account individually in the estimation of esds in distances, angles and torsion angles; correlations between esds in cell parameters are only used when they are defined by crystal symmetry. An approximate (isotropic) treatment of cell esds is used for estimating esds involving l.s. planes.
Refinement. Refinement of F^2^ against ALL reflections. The weighted R-factor wR and goodness of fit S are based on F^2^, conventional R-factors R are based on F, with F set to zero for negative F^2^. The threshold expression of F^2^ > 2sigma(F^2^) is used only for calculating R-factors(gt) etc. and is not relevant to the choice of reflections for refinement. R-factors based on F^2^ are statistically about twice as large as those based on F, and R- factors based on ALL data will be even larger.

### Fractional atomic coordinates and isotropic or equivalent isotropic displacement parameters (Å^2^)

**Table:**

	*x*	*y*	*z*	*U*_iso_*/*U*_eq_	
Cu1	0.09853 (3)	0.81795 (2)	0.437142 (18)	0.02327 (10)	
N1	−0.0413 (2)	0.87989 (18)	0.31321 (14)	0.0236 (3)	
N2	−0.1026 (2)	0.70465 (19)	0.49414 (14)	0.0248 (3)	
N3	−0.1258 (2)	0.60892 (19)	0.59384 (14)	0.0262 (3)	
N4	0.2029 (2)	0.77149 (19)	0.57510 (14)	0.0243 (3)	
O1	−0.3452 (2)	0.63031 (17)	0.43908 (13)	0.0302 (3)	
O2	0.2363 (2)	0.97722 (17)	0.34524 (13)	0.0316 (3)	
O3	0.2579 (3)	1.1266 (2)	0.17829 (15)	0.0430 (4)	
C1	−0.1882 (3)	0.8163 (2)	0.31567 (17)	0.0243 (4)	
C2	−0.2883 (3)	0.8560 (2)	0.22629 (18)	0.0293 (4)	
H2B	−0.3903	0.8123	0.2263	0.035*	
C3	−0.2335 (3)	0.9632 (3)	0.13589 (18)	0.0331 (4)	
H3B	−0.3000	0.9923	0.0747	0.040*	
C4	−0.0801 (3)	1.0273 (2)	0.13613 (18)	0.0307 (4)	
H4	−0.0429	1.0991	0.0758	0.037*	
C5	0.0152 (3)	0.9817 (2)	0.22791 (17)	0.0261 (4)	
C6	−0.2228 (3)	0.7064 (2)	0.42312 (16)	0.0242 (4)	
C7	−0.0134 (3)	0.5975 (2)	0.66471 (17)	0.0274 (4)	
H7	−0.0378	0.5299	0.7307	0.049 (8)*	
C8	0.1460 (3)	0.6693 (2)	0.66302 (17)	0.0263 (4)	
C9	0.2380 (3)	0.6271 (3)	0.75670 (18)	0.0324 (4)	
H9A	0.1984	0.5561	0.8168	0.039*	
C10	0.3894 (3)	0.6908 (3)	0.7609 (2)	0.0357 (5)	
H10A	0.4529	0.6620	0.8232	0.043*	
C11	0.4442 (3)	0.7971 (3)	0.67170 (19)	0.0325 (5)	
H11A	0.5433	0.8431	0.6730	0.039*	
C12	0.3489 (3)	0.8337 (2)	0.58068 (18)	0.0287 (4)	
H12A	0.3869	0.9046	0.5200	0.034*	
C13	0.1842 (3)	1.0349 (2)	0.25009 (18)	0.0287 (4)	
O4	0.3293 (2)	0.63057 (18)	0.35862 (13)	0.0264 (3)	
O5	0.3746 (3)	0.7227 (2)	0.12947 (18)	0.0412 (4)	
O6	0.7331 (3)	0.6617 (2)	0.00850 (16)	0.0414 (4)	
O7	0.1112 (3)	0.6122 (3)	0.06140 (17)	0.0461 (4)	
H4B	0.416 (5)	0.615 (3)	0.381 (2)	0.033 (8)*	
H7B	0.158 (5)	0.537 (4)	0.052 (3)	0.045 (9)*	
H6A	0.733 (4)	0.723 (3)	−0.041 (3)	0.033 (7)*	
H5B	0.371 (5)	0.693 (4)	0.188 (3)	0.043 (9)*	
H4A	0.291 (5)	0.566 (4)	0.381 (3)	0.045 (9)*	
H5A	0.303 (5)	0.707 (4)	0.113 (3)	0.044 (10)*	
H7A	−0.003 (6)	0.632 (4)	0.042 (3)	0.061 (10)*	
H6B	0.632 (5)	0.680 (4)	0.044 (3)	0.048 (9)*	

### Atomic displacement parameters (Å^2^)

**Table:**

	*U*^11^	*U*^22^	*U*^33^	*U*^12^	*U*^13^	*U*^23^
Cu1	0.02030 (15)	0.02569 (15)	0.02592 (15)	−0.00806 (10)	−0.00475 (9)	−0.00513 (10)
N1	0.0186 (7)	0.0252 (8)	0.0273 (8)	−0.0051 (6)	−0.0031 (6)	−0.0063 (6)
N2	0.0209 (8)	0.0282 (8)	0.0262 (8)	−0.0069 (6)	−0.0017 (6)	−0.0071 (6)
N3	0.0225 (8)	0.0269 (8)	0.0289 (8)	−0.0070 (6)	−0.0001 (6)	−0.0067 (6)
N4	0.0208 (8)	0.0287 (8)	0.0266 (8)	−0.0066 (6)	−0.0026 (6)	−0.0109 (6)
O1	0.0236 (7)	0.0330 (7)	0.0377 (8)	−0.0116 (6)	−0.0048 (6)	−0.0083 (6)
O2	0.0299 (8)	0.0300 (7)	0.0377 (8)	−0.0133 (6)	−0.0068 (6)	−0.0039 (6)
O3	0.0413 (9)	0.0402 (9)	0.0452 (10)	−0.0197 (8)	−0.0075 (8)	0.0071 (7)
C1	0.0200 (9)	0.0239 (9)	0.0292 (9)	−0.0023 (7)	−0.0033 (7)	−0.0088 (7)
C2	0.0251 (10)	0.0328 (10)	0.0336 (10)	−0.0056 (8)	−0.0074 (8)	−0.0123 (8)
C3	0.0342 (11)	0.0352 (11)	0.0297 (10)	−0.0021 (9)	−0.0108 (8)	−0.0083 (8)
C4	0.0317 (10)	0.0303 (10)	0.0274 (9)	−0.0041 (8)	−0.0022 (8)	−0.0058 (8)
C5	0.0243 (9)	0.0220 (9)	0.0303 (9)	−0.0035 (7)	−0.0018 (7)	−0.0062 (7)
C6	0.0187 (8)	0.0250 (9)	0.0290 (9)	−0.0039 (7)	−0.0005 (7)	−0.0093 (7)
C7	0.0278 (10)	0.0276 (9)	0.0260 (9)	−0.0082 (8)	−0.0006 (7)	−0.0044 (7)
C8	0.0240 (9)	0.0270 (9)	0.0286 (9)	−0.0033 (7)	−0.0029 (7)	−0.0108 (7)
C9	0.0335 (11)	0.0349 (11)	0.0286 (10)	−0.0062 (9)	−0.0065 (8)	−0.0067 (8)
C10	0.0309 (11)	0.0435 (12)	0.0375 (11)	−0.0035 (9)	−0.0125 (9)	−0.0167 (10)
C11	0.0236 (10)	0.0387 (11)	0.0405 (12)	−0.0045 (8)	−0.0061 (8)	−0.0197 (9)
C12	0.0229 (9)	0.0324 (10)	0.0346 (10)	−0.0076 (8)	−0.0026 (8)	−0.0138 (8)
C13	0.0240 (9)	0.0249 (9)	0.0368 (11)	−0.0071 (7)	−0.0034 (8)	−0.0048 (8)
O4	0.0221 (7)	0.0270 (8)	0.0315 (7)	−0.0078 (6)	−0.0043 (6)	−0.0059 (6)
O5	0.0384 (10)	0.0548 (11)	0.0350 (10)	−0.0198 (8)	−0.0031 (8)	−0.0091 (8)
O6	0.0392 (10)	0.0409 (10)	0.0341 (9)	−0.0032 (8)	−0.0033 (8)	0.0018 (8)
O7	0.0441 (11)	0.0474 (11)	0.0530 (11)	−0.0105 (9)	−0.0160 (9)	−0.0150 (9)

### Geometric parameters (Å, º)

**Table:**

Cu1—N1	1.9042 (17)	C4—C5	1.375 (3)
Cu1—N4	1.9325 (17)	C4—H4	0.9300
Cu1—N2	1.9415 (17)	C5—C13	1.525 (3)
Cu1—O2	2.0084 (15)	C7—C8	1.470 (3)
Cu1—O4	2.3379 (15)	C7—H7	0.9300
N1—C5	1.326 (3)	C8—C9	1.384 (3)
N1—C1	1.336 (2)	C9—C10	1.388 (3)
N2—C6	1.353 (3)	C9—H9A	0.9300
N2—N3	1.360 (2)	C10—C11	1.376 (4)
N3—C7	1.283 (3)	C10—H10A	0.9300
N4—C12	1.349 (3)	C11—C12	1.373 (3)
N4—C8	1.348 (3)	C11—H11A	0.9300
O1—C6	1.232 (2)	C12—H12A	0.9300
O2—C13	1.270 (3)	O4—H4B	0.70 (3)
O3—C13	1.228 (3)	O4—H4A	0.70 (4)
C1—C2	1.373 (3)	O5—H5B	0.72 (3)
C1—C6	1.506 (3)	O5—H5A	0.65 (3)
C2—C3	1.389 (3)	O6—H6A	0.74 (3)
C2—H2B	0.9300	O6—H6B	0.78 (4)
C3—C4	1.389 (3)	O7—H7B	0.72 (3)
C3—H3B	0.9300	O7—H7A	0.86 (4)
			
N1—Cu1—N4	170.94 (7)	N1—C5—C4	119.82 (19)
N1—Cu1—N2	80.89 (7)	N1—C5—C13	111.31 (18)
N4—Cu1—N2	95.11 (7)	C4—C5—C13	128.86 (19)
N1—Cu1—O2	80.84 (7)	O1—C6—N2	127.17 (19)
N4—Cu1—O2	101.87 (7)	O1—C6—C1	121.94 (18)
N2—Cu1—O2	160.31 (7)	N2—C6—C1	110.88 (16)
N1—Cu1—O4	91.72 (6)	N3—C7—C8	133.21 (19)
N4—Cu1—O4	96.87 (6)	N3—C7—H7	113.4
N2—Cu1—O4	97.22 (7)	C8—C7—H7	113.4
O2—Cu1—O4	90.63 (6)	N4—C8—C9	120.50 (19)
C5—N1—C1	123.44 (18)	N4—C8—C7	122.52 (18)
C5—N1—Cu1	118.13 (14)	C9—C8—C7	116.98 (19)
C1—N1—Cu1	118.41 (14)	C8—C9—C10	120.0 (2)
C6—N2—N3	114.57 (16)	C8—C9—H9A	120.0
C6—N2—Cu1	117.02 (13)	C10—C9—H9A	120.0
N3—N2—Cu1	127.98 (13)	C11—C10—C9	119.1 (2)
C7—N3—N2	118.37 (17)	C11—C10—H10A	120.4
C12—N4—C8	118.86 (18)	C9—C10—H10A	120.4
C12—N4—Cu1	118.88 (14)	C12—C11—C10	118.3 (2)
C8—N4—Cu1	122.02 (14)	C12—C11—H11A	120.8
C13—O2—Cu1	114.80 (13)	C10—C11—H11A	120.8
N1—C1—C2	119.63 (19)	N4—C12—C11	123.1 (2)
N1—C1—C6	112.24 (17)	N4—C12—H12A	118.4
C2—C1—C6	128.12 (19)	C11—C12—H12A	118.4
C1—C2—C3	118.32 (19)	O3—C13—O2	125.5 (2)
C1—C2—H2B	120.8	O3—C13—C5	119.8 (2)
C3—C2—H2B	120.8	O2—C13—C5	114.64 (18)
C2—C3—C4	120.6 (2)	Cu1—O4—H4B	108 (2)
C2—C3—H3B	119.7	Cu1—O4—H4A	103 (3)
C4—C3—H3B	119.7	H4B—O4—H4A	106 (3)
C5—C4—C3	118.2 (2)	H5B—O5—H5A	108 (4)
C5—C4—H4	120.9	H6A—O6—H6B	107 (3)
C3—C4—H4	120.9	H7B—O7—H7A	105 (3)
			
N4—Cu1—N1—C5	112.0 (4)	C1—C2—C3—C4	0.4 (3)
N2—Cu1—N1—C5	176.41 (15)	C2—C3—C4—C5	0.0 (3)
O2—Cu1—N1—C5	3.82 (14)	C1—N1—C5—C4	0.4 (3)
O4—Cu1—N1—C5	−86.55 (15)	Cu1—N1—C5—C4	178.88 (15)
N4—Cu1—N1—C1	−69.4 (4)	C1—N1—C5—C13	179.22 (17)
N2—Cu1—N1—C1	−5.02 (14)	Cu1—N1—C5—C13	−2.3 (2)
O2—Cu1—N1—C1	−177.62 (15)	C3—C4—C5—N1	−0.5 (3)
O4—Cu1—N1—C1	92.02 (15)	C3—C4—C5—C13	−179.05 (19)
N1—Cu1—N2—C6	7.05 (14)	N3—N2—C6—O1	−1.1 (3)
N4—Cu1—N2—C6	178.85 (14)	Cu1—N2—C6—O1	171.93 (16)
O2—Cu1—N2—C6	29.2 (3)	N3—N2—C6—C1	179.46 (15)
O4—Cu1—N2—C6	−83.54 (14)	Cu1—N2—C6—C1	−7.5 (2)
N1—Cu1—N2—N3	179.04 (16)	N1—C1—C6—O1	−176.13 (17)
N4—Cu1—N2—N3	−9.16 (16)	C2—C1—C6—O1	4.7 (3)
O2—Cu1—N2—N3	−158.79 (17)	N1—C1—C6—N2	3.3 (2)
O4—Cu1—N2—N3	88.45 (16)	C2—C1—C6—N2	−175.89 (18)
C6—N2—N3—C7	178.04 (17)	N2—N3—C7—C8	0.1 (3)
Cu1—N2—N3—C7	5.9 (3)	C12—N4—C8—C9	−1.0 (3)
N1—Cu1—N4—C12	−113.4 (4)	Cu1—N4—C8—C9	173.31 (15)
N2—Cu1—N4—C12	−176.78 (15)	C12—N4—C8—C7	179.16 (18)
O2—Cu1—N4—C12	−6.80 (16)	Cu1—N4—C8—C7	−6.5 (3)
O4—Cu1—N4—C12	85.30 (15)	N3—C7—C8—N4	0.5 (4)
N1—Cu1—N4—C8	72.3 (4)	N3—C7—C8—C9	−179.4 (2)
N2—Cu1—N4—C8	8.91 (16)	N4—C8—C9—C10	0.4 (3)
O2—Cu1—N4—C8	178.88 (15)	C7—C8—C9—C10	−179.80 (19)
O4—Cu1—N4—C8	−89.02 (15)	C8—C9—C10—C11	0.9 (3)
N1—Cu1—O2—C13	−4.88 (15)	C9—C10—C11—C12	−1.4 (3)
N4—Cu1—O2—C13	−176.09 (14)	C8—N4—C12—C11	0.5 (3)
N2—Cu1—O2—C13	−27.1 (3)	Cu1—N4—C12—C11	−174.03 (15)
O4—Cu1—O2—C13	86.76 (15)	C10—C11—C12—N4	0.7 (3)
C5—N1—C1—C2	0.1 (3)	Cu1—O2—C13—O3	−175.11 (19)
Cu1—N1—C1—C2	−178.37 (14)	Cu1—O2—C13—C5	4.9 (2)
C5—N1—C1—C6	−179.18 (17)	N1—C5—C13—O3	178.1 (2)
Cu1—N1—C1—C6	2.3 (2)	C4—C5—C13—O3	−3.2 (3)
N1—C1—C2—C3	−0.5 (3)	N1—C5—C13—O2	−1.9 (3)
C6—C1—C2—C3	178.64 (18)	C4—C5—C13—O2	176.8 (2)

### Hydrogen-bond geometry (Å, º)

**Table:**

*D*—H···*A*	*D*—H	H···*A*	*D*···*A*	*D*—H···*A*
O4—H4*B*···O1^i^	0.70 (3)	2.04 (3)	2.718 (2)	165 (3)
O7—H7*B*···O6^ii^	0.72 (3)	2.09 (3)	2.796 (3)	167 (3)
O6—H6*A*···O3^iii^	0.74 (3)	1.94 (3)	2.675 (3)	176 (3)
O5—H5*B*···O4	0.72 (3)	2.07 (3)	2.788 (3)	173 (3)
O4—H4*A*···N3^iv^	0.70 (4)	2.20 (4)	2.878 (2)	163 (3)
O4—H4*A*···O1^iv^	0.70 (4)	2.56 (3)	3.053 (2)	129 (3)
O5—H5*A*···O7	0.65 (3)	2.10 (4)	2.742 (3)	168 (4)
O7—H7*A*···O6^v^	0.86 (4)	1.95 (4)	2.803 (3)	175 (3)
O6—H6*B*···O5	0.78 (4)	1.94 (4)	2.718 (3)	178 (3)

Symmetry codes: (i) *x*+1, *y*, *z*; (ii) −*x*+1, −*y*+1, −*z*; (iii) −*x*+1, −*y*+2, −*z*; (iv) −*x*, −*y*+1, −*z*+1; (v) *x*−1, *y*, *z*.
